# Large Animal Models of Heart Failure: A Translational Bridge to Clinical Success

**DOI:** 10.1016/j.jacbts.2020.04.011

**Published:** 2020-08-24

**Authors:** Kleiton Augusto Santos Silva, Craig A. Emter

**Affiliations:** Department of Biomedical Sciences, University of Missouri-Columbia, Columbia, Missouri

**Keywords:** heart failure, HFpEF, HFrEF, large animal model, preclinical, AF, atrial fibrillation, ECM, extracellular matrix, EDP, end-diastolic pressure, EF, ejection fraction, FDA, Food and Drug Administration, HF, heart failure, HFpEF, heart failure with preserved ejection fraction, HFrEF, heart failure with reduced ejection fraction, I/R, ischemia/reperfusion, IABP, intra-aortic balloon pump, LAD, left anterior descending, LCx, left circumflex, LV, left ventricular, MI, myocardial infarction, PCI, percutaneous coronary intervention, RV, right ventricular

## Abstract

Preclinical large animal models of heart failure (HF) play a critical and expanding role in translating basic science findings to the development and clinical approval of novel therapeutics and devices. The complex combination of cardiovascular events and risk factors leading to HF has proved challenging for the development of new treatments for these patients. This state-of-the-art review presents historical and recent studies in porcine, ovine, and canine models of HF and outlines existing methodologies and physiological phenotypes. The translational importance of large animal studies to clinical success is also highlighted with an overview of recent devices approved by the Food and Drug Administration, together with preclinical HF animal studies used to aid both development and safety and/or efficacy testing. Increasing the use of large animal models of HF holds significant potential for identifying the novel mechanisms underlying the clinical condition and to improving physiological and economical translation of animal research to successfully treat human HF.

## Highlights

• Preclinical large animal models play a critical and expanding role in translating basic science findings to the development and clinical approval of novel cardiovascular therapeutics.
• This state-of-the-art review outlines existing methodologies and physiological phenotypes of several HF models developed in large animals. A comprehensive list of porcine, ovine, and canine models of disease are presented, and the translational importance of these studies to clinical success is highlighted through a brief overview of recent devices approved by the FDA alongside associated clinical trials and preclinical animal reports.
• Increasing the use of large animal models of HF holds significant potential for identifying new mechanisms underlying this disease and providing valuable information regarding the safety and efficacy of new therapies, thus, improving physiological and economical translation of animal research to the successful treatment of human HF.

The complexity of heart failure (HF) has challenged the scientific community for decades. Multifaceted signatures of pathophysiological mechanisms driving HF are under intense investigation. However, the heterogeneous nature of the disease has limited therapeutic advances in the field. Not surprisingly, the prevalence of HF continues to increase at an alarming rate. Currently, it is estimated that 6.5 million people in United States have HF; by 2030, HF will affect >8 million people (1). In addition, HF negatively affects the economy, costing several billions of dollars each year (≈$70 billion by 2030).

A defining characteristic of HF is the inability of the heart to pump enough blood to the body, which leads to poor quality of life for patients with this condition. In the past 30 years, the diagnosis of HF has evolved 2 primary categories: 1) HF with reduced ejection fraction (HFrEF), characterized by a resting ejection fraction (EF) of ≤40% and traditionally referred to as systolic HF; and 2) HF with preserved ejection fraction (HFpEF), characterized by a resting EF of ≥50% and traditionally referred to as diastolic HF (2,3). Recently, a third category of HF was introduced to the field, referred to as HF with midrange EF, characterized by a resting EF range from 40% to 50% (4).

The combination of numerous risk factors (physical inactivity), comorbidities (obesity, hypertension, type 2 diabetes, chronic kidney disease), and disease modifiers (age, sex) associated with HF has made improving therapeutic options for treating the overall syndrome difficult. Contributing to these difficulties is the lack of ideal animal models that reliably replicate most of the pathophysiological features often found in human HF. Large animal models of HF (e.g., pigs, sheep, etc.) have some advantages in terms of clinical translation given key determinants of myocardial work and energy consumption, such as left ventricular (LV) wall tension, heart rate, and vascular wall-to-lumen ratios are more similar to humans (5, 6, 7, 8, 9, 10, 11, 12, 13). Thus, it could be argued that the use of preclinical large animal models of HF for the discovery of novel mechanisms underlying the syndrome and development and/or testing of new therapeutic options for the treatment of HF are not only warranted, but necessary to advance our understanding of this highly prevalent cardiovascular disease.

This state-of-the-art review outlines existing methodologies and physiological phenotypes of several HF models developed in large animals (Central Illustration). Historical and recent studies of HF in porcine, ovine, and canine models of disease are presented in Table 1. The translational importance of large animal studies to clinical success is also highlighted, providing a brief overview of recent devices approved by the U.S. Food and Drug Administration (FDA) alongside their associated clinical trials and preclinical animal studies.

**Central Illustration:**
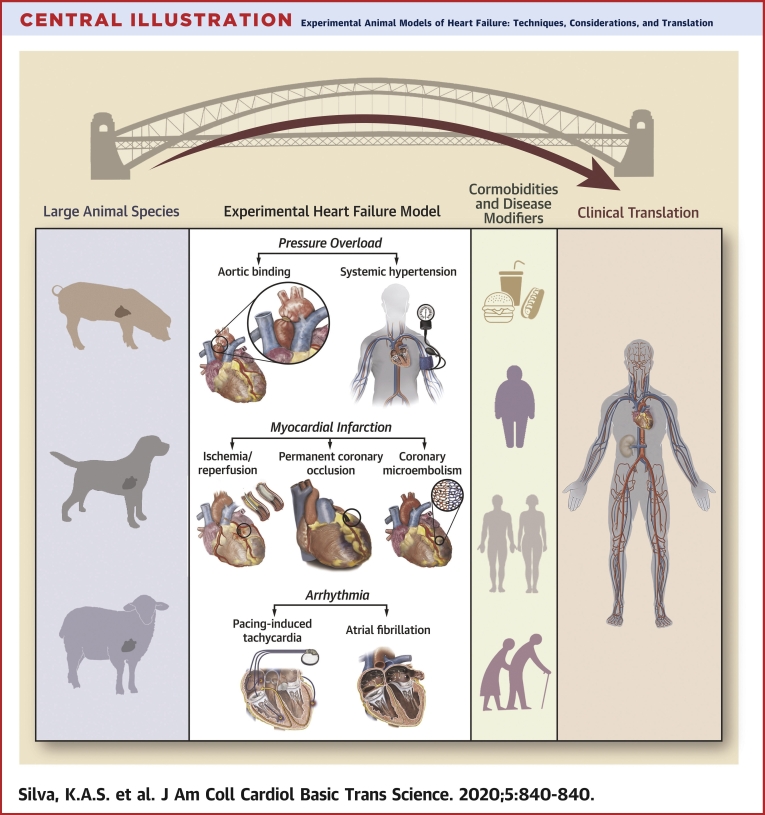
Experimental Animal Models of Heart Failure: Techniques, Considerations, and Translation

**Table 1:** Summary of Large Animal HF Studies Highlighting Interventions, Techniques, General Function and Species

Model of Heart Failure	LVEF	Type of Animal (Ref. #)
Method		
Pressure overload		
Aortic banding	Preserved	Pig (21, 22, 23, 24, 25, 26, 27, 28, 29, 30, 31, 32, 33, 34, 35)
	Reduced	Sheep (47, 48, 49, 50, 51, 52)
	Preserved or reduced	Dog (54, 55, 56, 57, 58)
Renal wrapping or embolization	Preserved	Dog (59, 60, 61, 62, 63, 64); pig (65)
DOCA	Preserved	Pig (66,67)
Myocardial infarction		
Ischemia/reperfusion	Reduced	Dog (86); pig (87,88,111); sheep (82, 83, 84, 85, 86, 87, 88, 89, 90, 91, 92)
Permanent coronary occlusion	Reduced	Pig (93, 94, 95, 96, 97, 98, 99, 100); sheep (89,101,102)
Coronary microembolization	Reduced	Dog (103,104); pig (105, 106, 107); sheep (108, 109, 110)
Arrhythmia		
Pacing-induced tachycardia	Reduced	Dog (118,119); pig (120,121,124); sheep (122,123)
Atrial fibrillation	Preserved or reduced	Dog (125,127); pig (125,126); sheep (128)

DOCA = deoxycorticosterone acetate; HF = heart failure; LVEF = left ventricular ejection fraction.

## HF Induced by Pressure Overload

Chronic pressure overload resulting from aortic valve stenosis or systemic hypertension may ultimately lead to HF (14,15). Over time, sustained increases in myocardial work required to overcome chronic elevations in afterload can induce structural, physiological, and molecular changes that result in pathological cardiac remodeling (16, 17, 18). In addition, vascular dysfunction in numerous organs, including the heart, brain, skeletal muscle, and renal systems, are negatively affected and may further contribute to cardiovascular dysfunction. To maintain normal function (often measured as EF), the heart transitions to a compensated stage characterized by concentric LV hypertrophy and increased myocardial stiffness associated with decreased myocardial relaxation, increased LV filling pressure, pulmonary congestion, and decreased cardiac reserve (16,17,19,20). Patients who have transitioned to a compensated stage of function often show signs and symptoms similar to those observed in HFpEF.

Given the significant number of HF patients with antecedent hypertension and/or aortic stenosis, numerous large animal models of pressure overload−induced HF have been developed to enhance our understanding of how these pathological mechanisms contribute to disease development. Many of these models have incorporated parallel comorbidities, such as obesity, type 2 diabetes, and chronic kidney disease, into the overall design in an effort to more comprehensively imitate the clinical syndrome, alongside traditional physiological features of HF (e.g., pulmonary congestion, dyspnea, and exercise intolerance). As a result, animal models of experimental pressure overload−induced HF have been developed using surgical techniques such as transthoracic aortic constriction (i.e., aortic banding), renal wrapping, and renal microembolization in pigs, sheep, and dogs. Endocrine-mediated methods based on high-salt diets like deoxycorticosterone acetate have also been used. Transthoracic aortic constriction methods attempt to recreate aortic stenosis by narrowing the aorta, which results in both local increases in myocardial afterload and neurohumoral involvement; the severity of each depends on the location of the aortic constriction (e.g., ascending aorta vs. the descending aorta). Aortic banding increases the LV aortic pressure gradient, induces concentric LV hypertrophy, increases myocardial stiffness, and impairs myocardial relaxation similar to that observed in aortic stenosis. However, aortic banding fails to recapitulate calcification and fibrotic lesions in the aortic valve or significant increases in vascular stiffness occurring along the length of the aorta as often seen in human aortic stenosis. Renal wrapping, renal microembolization, and implantation of deoxycorticosterone acetate pellets induce systemic hypertension via neurohumoral activation. Although effective, these methods are limited by their inability to incorporate genetic factors that often contribute to developing hypertension and use supra-physiological doses of salt that may also have disproportionate impacts on neurogenic and neurohormonal activation. With these general strengths and weakness in mind, the following sections discuss existing large animal models of pressure overload−induced HF and highlight the physiological and molecular phenotypes associated with each.

### Aortic banding models

Several different studies have examined aortic banding in swine in the absence of comorbidities. Cardiac pressure overload was induced by constricting the ascending aorta in 45-day-old Yorkshire pigs using a 60- to 70-mm Hg systolic pressure gradient over 2 months (21, 22, 23). Traditional experimental signs of HF in these animals included peritoneal ascites in the range of 100 to 2,000 ml in less than one-half of all aortic-banded animals. Signs of both LV and right ventricular (RV) hypertrophy were observed in parallel with diastolic dysfunction evident as increased end-diastolic pressure (EDP) depending on the severity of disease. This model demonstrated significant impairments to myocardial oxidative and high-energy phosphate bioenergetics measured using primarily nuclear magnetic resonance spectroscopy techniques.

**Figure 1:**
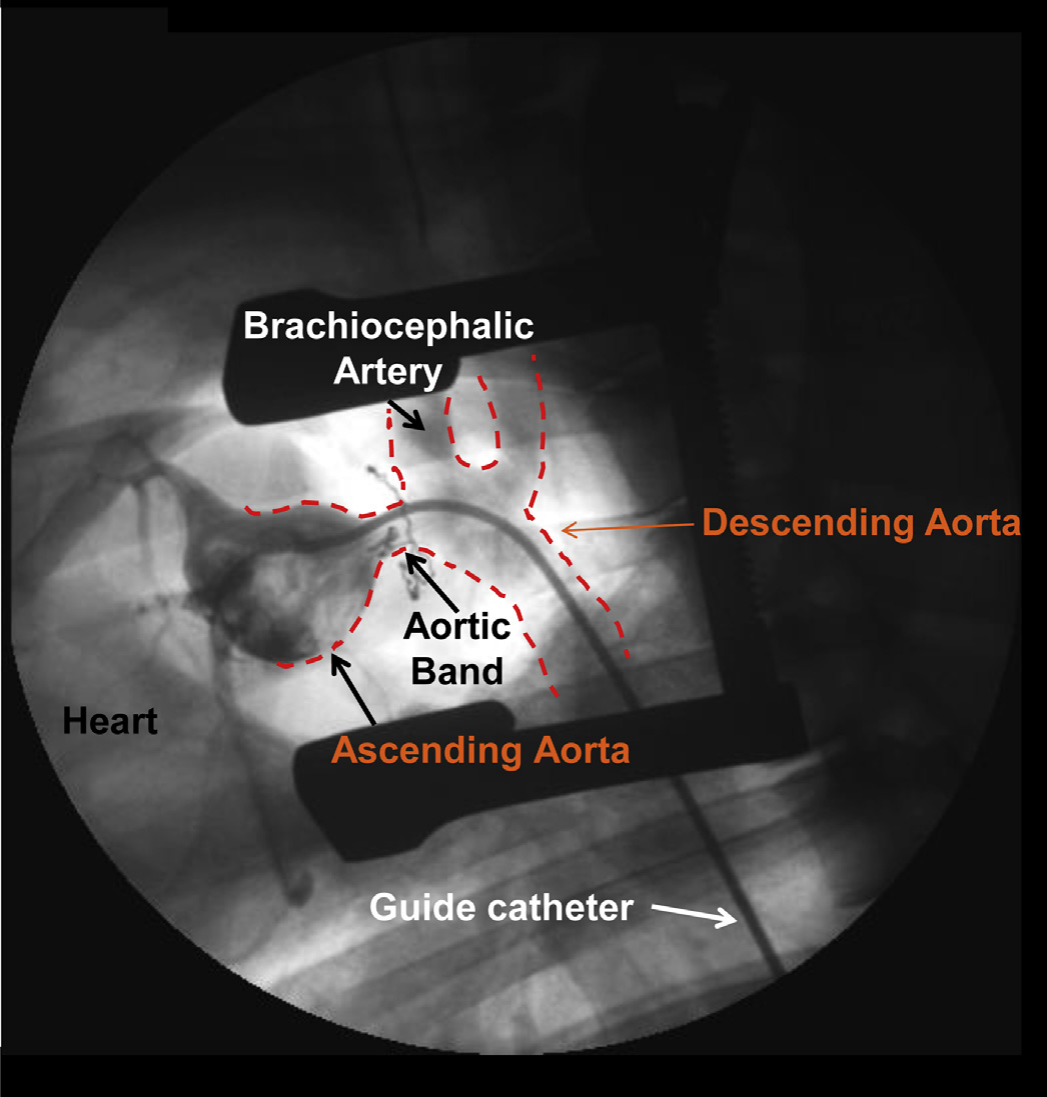
Representative Angiogram Illustrating Aortic Banding Technique for Pressure Overload-Induced HF Angiography from a male Yucatan mini-pig (8 months old) showing placement of the aortic band (anatomically marked by radiopaque umbilical tape) and narrowing of the ascending aorta proximal to the brachiocephalic artery (large peripheral vessels outlined by **red dashed line**). The systolic pressure gradient is determined by a fluid-filled guide catheter (femoral insertion) connected to a pressure transducer and measured proximally and/or distally to the banding location. HF = heart failure.

Aortic banding was also used to induce chronic pressure overload−induced HF in both 3- and 8-month-old Yucatan miniature swine using a 50- or 70-mm Hg systolic pressure gradient, respectively, placed on the ascending aorta over 6 months (see Figure 1 for surgical visualization) (24, 25, 26, 27, 28, 29, 30, 31, 32). In this model, classic signs of experimental HF included increased LV brain natriuretic peptide mRNA levels and lung weight. Molecular and physiological phenotypes were most reminiscent of HFpEF in these animals, including global concentric hypertrophy, normal resting EF, diastolic dysfunction (increased end-diastolic pressure−volume relationship, impaired diastolic strain during both early and late diastole, altered cardiomyocyte calcium handling), increased fibrosis and altered regulation of the extracellular matrix (ECM), mitochondrial dysfunction, and significant sex-based disparities in disease manifestation. This model also exhibited signs of significant vascular dysfunction in both coronary and peripheral vascular beds, including the brain, in which significant cerebrovascular impairment was observed alongside cardiogenic dementia.

Other models of HF developed by using aortic banding in pigs included studies by Ishikawa et al. (33) and Yarbrough et al. (34). A customized rubber band with a fixed inner radius of 12 cm was placed on the ascending aorta of Yorkshire pigs (10 to 13 kg) that were subsequently followed for 3 to 5 months by Ishikawa et al. (33). These animals did not show historical experimental signs of HF, but did demonstrate preserved EF, diastolic dysfunction (increased end-diastolic pressure−volume relationship and increased EDP with pacing), and LV hypertrophy with increased fibrosis. Using an inflatable cuff placed on the ascending aorta of Yorkshire pigs, Yarbrough et al. (34) progressively narrowed the ascending aorta by inflating the cuff weekly over 5 weeks. Final measurements showed a pressure gradient of 66 mm Hg with diastolic dysfunction (increased LV EDP and Tau) and increased fibrosis associated with regional myocardial stiffness and altered levels of ECM regulatory biomarkers (MMP-7 and -14, TIMP-1 and -4). This more acute model of myocardial pressure overload also did not show traditional indicators of HF.

A more recent attempt investigated the heterogeneous aspects of HF by including comorbidities via a combination of Western diet (10 months) and chronic pressure overload using aortic banding (6 months; 70-mm Hg systolic pressure gradient) in female Ossabaw swine (35), a unique translational large animal model genetically predisposed to obesity and metabolic derangement that does not develop HF from dietary intervention alone (36, 37, 38, 39, 40, 41, 42, 43). Recently listed as a multihit model useful for examining the heterogenous nature of HFpEF by the National Heart, Lung, and Blood Institute HFpEF working group (44), these animals displayed classic experimental markers of HF, including increased lung weight and genetic signatures that indicated the induction of numerous HF-related genes (e.g., natriuretic peptides). Significant inflammation and metabolic derangement (obesity, insulin resistance, dyslipidemia) was observed at both clinical and molecular levels, which has been considered a major causative component of HFpEF (45,46). Molecular and physiological phenotypes were also evocative of HFpEF, including concentric LV hypertrophy, normal EF, diastolic dysfunction (increased end-diastolic pressure−volume relationship, impaired diastolic strain during both early and late diastole, titin isoform shift, altered cardiomyocyte calcium handling), changes in the composition of ECM, and mitochondrial dysfunction. Biomarkers with potential relevance to HFpEF (e.g., Pentraxin-3 and interleukin 1 receptor-like 1) were also observed in parallel with significant microvascular dysfunction in coronary and peripheral (skeletal muscle, brain) vascular beds (35).

In contrast to swine models, the development of systolic dysfunction tends to precede diastolic dysfunction in ovine models of pressure overload−induced HF. Aortic banding of the ascending aorta in sheep was accomplished using a number of different methods, varying from static banding in young animals (47) to adjustable inflatable occluders in adult animals age 6 months to 2 years (47, 48, 49, 50, 51, 52), which resulted in a wide range of systolic pressure gradients measuring 25 to 80 mm Hg. Demonstration of experimental HF was typically presented as a decrease in fractional shortening or EF, which indicated pressure overload in sheep might be more representative of HFrEF. Two of these studies addressed the often asked but understudied question, “What happens to the heart if the cardiac pressure overload is removed?” (49,52). These studies demonstrated some plasticity for the myocardium to return to normal via reversal of pathological cardiac remodeling, numerous cellular markers of apoptosis, and ECM regulation and modification.

Acute and chronic models of canine pressure overload-induced HF have been heavily used for >40 years. The use of dog models has diminished over time in part due to its extensive collateral circulation in dogs, which differs dramatically from that of humans and other large animals (e.g., pigs) (53). We referenced several historical studies that provide a foundation for functional and structural adaptations to the myocardium and coronary vasculature in aortic banded dogs (54, 55, 56, 57, 58).

### Systemic hypertension models

Historical studies led to the development of canine models of systemic hypertension by clamping of a renal artery (59) and wrapping 1 or 2 kidneys in cellophane (60). These models of systemic pressure overload have been used to study several aspects of HFpEF in both young and importantly, old dogs, given the significant aging component associated with this type of HF (45). Use of the “Page model” of bilateral renal wrapping for 6 to 12 weeks (both with and without deoxycorticosterone acetate) induced hypertension with systolic blood pressure reaching as high as 250 mm Hg, concentric LV remodeling, preserved EF, diastolic dysfunction, and fibrosis (61, 62, 63, 64). Classic signs of experimental HF were mostly absent, and comorbidities often associated with HFpEF were not incorporated into the model.

Two more recent swine models induced systemic pressure overload via hypertension using a combination of factors layered with comorbidities relevant to HFpEF. Sorop et al. (65) combined systemic hypertension (renal artery embolization), diabetes (streptozotocin), and hypercholesterolemia (high-fat diet) in 2- to 3-month-old Yorkshire-Landrace swine for 6 months. Renal embolization was achieved by injection of 75 mg of 38- to 42-μm polyethylene microspheres into the right kidney and into one-third of the left kidney. These animals, also listed by the National Heart, Lung, and Blood Institute HFpEF working group as a multihit model of HFpEF (44), demonstrated hemodynamic indicators of experimental HF, including increased left atrial pressure per a given cardiac index at rest and during exercise. Ejection fractio was more in the mid-range in this model (45%) and observed in parallel with diastolic dysfunction (increased end-diastolic pressure-volume relationship, titin isoform shift), increased LV collagen deposition, and coronary microvascular dysfunction. Obesity and concentric LV hypertrophy were absent, despite evidence of systemic increases in blood pressure, inflammation (plasma tumor necrosis factor−α), and metabolic derangement (type 1 diabetes, dyslipidemia).

The second of these studies included the combination of deoxycorticosterone acetate with Western diet for 12 weeks in Landrace swine (66). Hemodynamic markers of experimental HF were evident as a decrease in cardiac output and increase in LV EDP in response to pacing or dobutamine under anesthesia. Obesity, dyslipidemia, and increased systolic blood pressure were seen in combination with concentric LV hypertrophy, normal EF, diastolic dysfunction (titin isoform shift and altered phosphorylation), and LV nitric oxide synthase uncoupling. A separate magnetic resonance study in this same model also demonstrated impaired myocardial perfusion reserve, longitudinal strain, and torsion in response to dobutamine stress (67).

## HF Induced by Myocardial Infarction

Cardiac cell death associated with aberrant heart dysfunction is the main characteristic of a myocardial infarction (MI), which can ultimately lead to HF (68,69). This catastrophic event occurs due to interruption of blood flow to a discrete area of the myocardium that results from partial or complete occlusion of 1 or multiple coronary arteries. Unbalanced myocardial blood supply and demand can be spontaneously precipitated by coronary artery disease (e.g., atherosclerosis or thrombosis) or can occur during periprocedural revascularization surgery performed to revert spontaneous ischemia (e.g., percutaneous coronary intervention [PCI] or coronary artery bypass grafting) (68,69). The correct identification of different types of MI in response to ischemia is critical for optimizing patient treatment and is an important consideration for translational studies attempting to model acute MI and the subsequent development of HF. In this regard, the Universal Definition of MI was recently updated based on critical clinical projections driven by MI, including biomarkers (e.g., cardiac troponin levels), pathological features (e.g., edema, reduced glycogen content, and mitochondrial abnormalities), electrocardiography (e.g., new ST-segment elevations), and imaging by echocardiography, radionuclide imaging, or resonance magnetic imaging (e.g., myocardial free wall rupture and mitral regurgitation) (70).

Cardiac structural, functional, and metabolic characterization after MI reveal disruption of the contractile apparatus, mitochondrial impairment, endothelial dysfunction, and increased cell death (71,72). Coronary revascularization procedures such as PCI or coronary artery bypass grafting can improve survival rate post-MI and quality of life, but often result in the development of decompensated HF (i.e., HFrEF) (73,74). Consistent with this progression of HF, animal models of MI-induced HF are characterized by an initial ischemic event followed by a decrease in cardiac output and reduced EF, ventricular dilation associated with normal or reduced wall thickness (i.e., eccentric hypertrophy), areas of focal fibrosis in the ischemic area, activation of neurohormonal systems, and decreased cardiac reserve (17).

Experimental models of MI-induced HF include ischemia/reperfusion (I/R), non-reversible coronary occlusion induced by coronary ligation or ameroid constrictors, and coronary microembolization. Each technique incorporates clinical features that encompass central and peripheral modifications observed in patients with HFrEF caused by MI. An I/R approach acutely occludes coronary blood flow to the myocardium followed by reintroduction of blood flow to the ischemic area (75,76). Molecular mechanisms driving reperfusion injury are sudden arrhythmias, myocardial stunning caused by calcium overload and oxidative stress, as well as microvascular and endothelial dysfunction (74,77). This method has been most often used in the left anterior descending coronary artery (LAD) or left circumflex coronary artery (LCx) by reversible ligation or inflatable angioplasty balloon. Although historically dogs were used in I/R studies, an extensive coronary collateral circulation present in the canine heart has significantly decreased the use of this model. In pigs and sheep, strengths of I/R approaches include the ability to create infarcts of a predictable size and location by taking advantage of similarities more reminiscent of human coronary arteries, including gross anatomical structure and an absence of existing collateral vessels. Weaknesses of swine and ovine I/R models include significant acute susceptibility to arrhythmia and difficulty imaging the heart using ultrasound techniques due to ruminant-dependent differences in gastrointestinal anatomy.

Non-reversible coronary occlusion is performed by suture ligation or ameroid constrictor placement without reperfusion. The coronary ligation by suture is an immediate approach to develop acute MI, whereas ameroid constrictors can mimic MI resulting from coronary stenosis due to progressive atherosclerotic plaque formation. Limitations to this technique include permanent occlusion of vascular flow to the myocardium, which is rarely seen clinically because routine treatment includes reperfusion of the ischemic myocardium via PCI or coronary artery bypass grafting.

Finally, coronary microembolizations are sequential injections of microspheres that can be performed acutely and/or over time (78,79). Accumulation of atherosclerotic plaque debris in the coronary microcirculation increases the incidence of microembolization, varying from 20% to 79% (80), which can also result from PCI (81). Disruption of coronary atherosclerotic plaques by rupture, erosion, or calcific nodules can release harmful substances and potentially aggregate in the distal coronary microcirculation, which causes vasoconstriction, inflammation, and potential microinfarcts (79,82). Currently, clinical evidence of atherosclerosis driving reduction of myocardial blood flow and incidence of MI in the absence of significant coronary occlusion is classified as MI in the absence of coronary artery disease (83, 84, 85). Atherosclerotic plaque disruption and subsequent coronary microembolization can impair myocardium contractility and increase inflammation predominantly mediated by tumor necrosis factor−α, with sustained embolization resulting in repetitive events of thrombogenesis leading to MI. Although this technique can model the chronic effects of gradually increasing ischemia to the myocardium over time, consistency and reproducibility of infarcts can be difficult because of multiple embolization surgical procedures and a limited ability to control the extent of occlusion throughout the coronary vascular tree. Furthermore, this model results in multiple infarct and remodeling sites in the myocardium (in contrast to an individual area with a focused distinct injury) that can introduce variability and inconsistency to the assessment process. The following sections examine these models of MI-induced HF in large animals and outline physiological and molecular phenotypes relevant to each technique.

### I/R models

Occlusion times of coronary arteries from 30 to 180 min in duration have been shown to cause an ischemic insult significant enough to induce myocardium cell death (70,71,75). Given extensive coronary anastomosis has resulted in the decreased use of dogs to study MI and subsequent HF (53,76), a recent study investigated the effects of LV mechanical unloading after reversible LAD coronary ligation plus ligation of branches originating from the LCx coronary artery that potentially feed the LAD coronary area (86). With the stated goal of preventing potential influence of collateral circulation, coronaries were ligated for 180 min and then reperfused. Four weeks post-MI, this method produced an infarcted area of approximately 16% and a LVEF of approximately 40% in parallel with increased LV EDP, LV end-systolic volume, and N-terminal pro−B-type natriuretic peptide suggestive of HFrEF. This I/R approach provides a new alternative method that may help account for coronary collateralization in dog models of MI-induced HF.

In Yorkshire swine, differences in MI-induced HF following occlusion of the proximal LAD or LCx was examined using an I/R protocol produced by inflation of an intracoronary angioplasty balloon for 120 min (87). After 3 months, occlusion of the LAD produced an infarct size of approximately 14% in contrast to approximately 10% after occlusion of the LCx coronary artery. Increases in LV weight and impairments to LV mechanics (torsion and radial and/or circumferential strain, assessed via 2-dimesional speckle tracking echocardiography) was the same between groups, with a greater decrease in LVEF and increased end-systolic and end-diastolic volumes observed in the proximal LAD occluded group. Overall, the I/R protocol that used proximal occlusion of the LAD resulted in more severe disease, which suggested it may be a better preclinical model of MI-induced HF. Other studies in swine showed impaired calcium handling (decreased calcium transient amplitude and increased diastolic calcium levels) 14 weeks post-I/R using occlusion of the proximal LCx coronary artery for 2 h, which resulted in an EF of 39% (88).

Ovine models of I/R were also used to examine MI-induced HF. Charles et al. (89) examined I/R in Coopworth ewes using 90 min of occlusion by intracoronary balloon angioplasty placed between the first and second diagonals of the LAD coronary artery (89). Acute increases in natriuretic peptide level and cardiac troponin T (peak at 7 h post-MI) were observed alongside reduced EF (38%) 7 days post-MI, although LV dilation was not observed. In Dorset hybrid sheep, infarction size was dependent on time of ischemia (range 45 min to 6 h) with damage susceptibility significantly influenced by regional myocardial location (90). Six hours of ischemia progressively increased LV volume and decreased EF (27%) in Dorset hybrid sheep 12 weeks post-MI (91). Recent work demonstrated a decrease in EF (38%) 2 weeks post-MI following 120 min of occlusion in the LCx coronary artery using balloon occlusion (92).

### Non-reversible coronary occlusion models

van der Velden et al. (93) studied MI caused by non-reversible LCx artery ligation in 2- to 3-month-old Yorkshire-Landrace pigs. Three weeks post-MI, permanent ligation of the LCx artery increased heart weight, end-diastolic and end-systolic areas, and significantly decreased EF (35%). The sarcoplasmic/endoplasmic reticulum salcium-ATPase 2a (SERCA2a) protein level was decreased, and skinned cardiomyocytes demonstrated decreased maximal force generation and increased calcium sensitivity believed to be mediated by altered PKA phosphorylation of troponin I. A separate group of studies in Yorkshire swine (45 days old) demonstrated permanent ligation of the either the LCx or LAD coronary artery decreased EF (25% to 30%) and the bioenergetic reservoir measured by concentration of high-energy phosphate levels (phosphocreatine/adenosine triphosphate ratio) 4 to 8 weeks post-MI (94,95).

Ameroid constrictors were also used with and without comorbidities such as obesity and type 2 diabetes in swine to induce MI and HF. Early studies placed 2.0- to 2.5-mm ameroid constrictors around the LCx coronary artery, with gradual occlusion of blood flow resulting in a highly variable infarct size (5% to 37% of the LV) (96,97). Recently, an ameroid constrictor was placed on the LAD coronary artery for 4 weeks in obese Ossabaw swine, which resulted in infarct sizes of approximately 15% (98). Other studies that examined obese Ossabaw swine placed an ameroid constrictor around the LCx for 7 weeks, which resulted in a model of chronic ischemia as opposed to MI-induced HF because no infarcts were observed (99,100).

Ovine models of ischemic heart disease induced by ameroid constrictor were also used. Chekanov et al. (101) analyzed the effects a 3.5-mm ameroid constrictor placement in the LCx coronary artery. After 4 weeks, EF decreased to 49% and was associated with an increase in LV end-diastolic and end-systolic volumes. A separate set of studies in Coopworth ewes showed significant decreases in EF% (20% to 25%) 1 to 4 weeks post-surgery after occluding the LAD coronary artery using a thrombogenic coil in parallel with increased plasma circulating natriuretic peptides, cardiac troponin T, and creatine kinase (89,102).

### Coronary microembolization

Attempting to replicate acute MI and subsequent HF in a dog model of coronary microembolization, Franciosa et al. (103) acutely injected 100-μl glass microspheres (approximately 475 μm in diameter) into the LCx coronary artery of mongrel dogs and evaluated the animals at 1, 3, and 10 months. Ten months post-surgery, MI (23% average scar size) was associated with a significant decrease in cardiac output (103). Later studies used polystyrene latex microspheres (77 to 102 μm in diameter) sequentially injected into the LAD and LCx coronary arteries over 1 to 3 weeks (3 to 9 total coronary embolization procedures/animal) (104). Three months post-surgery, cardiac output and EF (21%) were decreased and associated with a transmural MI distributed throughout the LV, septum, and RV. Signs of HF included LV dilation (increased end-diastolic volume), increased pulmonary artery wedge pressure, and increased plasma atrial natriuretic peptide and/or norepinephrine.

A cardiac magnetic resonance study infused microbeads 100 to 300 μm in diameter into the LAD coronary artery of farm pigs (34 kg), which resulted in decreased EF (36%) 1 week after microembolization (105). In 4- to 5-month-old Yucatan mini-swine, Hanes et al. (106) acutely injected 2 ml of 90 μm polystyrene microspheres into the LAD coronary artery. This model was used in a follow-up study, which demonstrated decreased EF (45%) and LV dilation (increased end-diastolic volume) in addition to significant electrophysiological remodeling of numerous myocardial ion currents (106). Recent studies also used microspheres to induce acute MI by injecting 1 ml of polyvinyl-alcohol microspheres (45 to 150 μm in diameter) every 3 to 10 min for 45 min total in pigs (107).

In adult Merino Wether sheep (51 kg), an average of 5 embolization procedures was administered every 2 weeks using 90-μm polystyrene microspheres in both the LAD and LCx coronary arteries (108,109). Several indicators of HF were observed 26 weeks post-procedure, including decreased EF (27%), increased pulmonary capillary wedge pressure, and LV dilation and wall thinning (increased end-diastolic volume and decreased LV wall thickness, respectively). Monreal et al. (110) injected 0.5 ml of 90-μm fluorescence polystyrene microspheres into the LCx coronary artery in Dorsett cross sheep (44 kg). This approach successfully reduced EF (25%), caused LV dilation (increased end-diastolic volume), and increased mean pulmonary artery pressure 4 months post-intervention with sustained dysfunction present 2 years later.

### Combination I/R + coronary microembolization

Recent work combined I/R and coronary microembolization protocols in Yorkshire swine (38 to 43 kg) (111). In this model, the LAD coronary artery was occluded using intracoronary balloon angioplasty for 60 min followed by embolization via autologous thrombus injection and reperfusion. Decreased EF (<40%) and cardiac output were observed in parallel with increased LV end-diastolic volume and pulmonary capillary wedge pressure 1 week post-surgery. Scar size was significantly increased in these animals compared with 90 min of I/R alone.

## HF Induced by Arrhythmia

The pathological interaction between arrhythmia and HF is well established, increasing both the risk of developing HF and morbidity and/or mortality in established HF cases (112). Recently, arrhythmia-induced cardiomyopathy was proposed as a more inclusive way to examine the diverse impact of electrophysiological pathology to the overall HF syndrome (113). Sudden cardiac death is significant cause of mortality in HF, regardless of EF, and the role of tachycardia to developing HF has long been appreciated. In particular, supraventricular arrhythmias such as atrial fibrillation (AF) can increase HF risk 3-fold (114, 115, 116). Similar to the human syndrome, the development of HF in animal models of arrhythmia-induced cardiomyopathy include bi-ventricular dilation and decreased wall thickness, followed by a steady deterioration of cardiac output and EF over time, activation of neurohormonal systems, and significant impairment of cellular calcium homeostasis. Experimental models of arrhythmia-induced HF are characterized by periods of chronic rapid pacing denoted primarily by anatomical location of the pacemaker. For tachycardia models, the pacemaker is implanted in the RV or LV, whereas animal models of AF often stimulate pathological pacing of the myocardium from an atrial location. Interestingly, arrhythmia-induced models of HF models include almost complete recovery of myocardial function and structure upon termination of the pacing stimulus. Important considerations for these techniques include: 1) changes to myocardial structure and function, which can vary significantly within the same heart based on proximity to the pacemaker; and 2) development of HF directly related to pacing rate and duration. Furthermore, atrial pacing has demonstrated an inability to sustain chronic AF for longer than 2 to 8 weeks and often requires parallel dosing of traditional cardiac therapeutics, including β-blockers and/or cardiac glycosides. Because of the historical use of these models, we briefly highlight these approaches in the following sections.

### Pacing-induced tachycardia

Originally reported in 1962 (117), a significant number of canine (118,119), swine (120,121), and ovine (122,123) models of pacing-induced HF have been developed and used in both acute and chronic experimental settings (references provided for historical context). Cardiac pacing using heart rates across a spectrum of 120 to 260 beats/min have been routinely shown to induce symptoms comparable to HFrEF, including decreased EF, ventricular dilation and decreased ventricular wall thickness, pulmonary involvement, increased plasma expression of biomarkers (e.g., natriuretic peptides), and neurohumoral activation. Recently, Möllmann et al. (124) desynchronized heart beats in swine (10 to 12 weeks old; 34 kg) using different pacing locations in the RV (110 beat/min for each lead, effective heart rate of 220 beats/min). Desynchronization caused more severe HF compared with single-lead pacing, which was characterized by significantly impairing LV systolic function (decreased cardiac and/or stroke volume index and fractional shortening), increased pulmonary capillary wedge pressure and LV end-diastolic dimension, cardiac hypertrophy, and pathological ECM remodeling.

### Atrial fibrillation

Although several large animal models of AF have been developed, studies in which this common arrhythmia results in HF are limited. Dosdall et al. (125) examined the impact of chronic rapid atrial pacing in mixed breed hounds, Boer and mixed breed goats, and Yorkshire swine in a study designed to determine the appropriate animal model to optimize long-term development of AF and subsequent HF. A pacemaker implanted in the right atria was programmed to stimulate at 50 Hz for 1 s followed by 1 s of no stimulation at 2 to 3 times the diastolic pacing threshold. Every 1 to 2 weeks, the pacemaker was deactivated to determine whether sustained AF had developed. If AF was sustained, the pacemaker was reprogrammed to stimulate AF only if the animal returned to normal sinus rhythm. Six months post-intervention, dogs were the only animals to develop signs of HF evident as a decrease in EF (33%) and increased LV fibrosis. Both pigs and goats failed to develop HF. Rapid atrial pacing was shown to induce HF in domestic swine and dogs (evident as a decrease in EF to ≈30%) 3 weeks or 3 months after the initiation of pacing, respectively (126,127). In sheep, the combination of atrial and RV pacing impaired LV contractility and relaxation during exercise (±dP/dt_max_) and increased biomarkers of HF (brain natriuretic peptide, endothelin) 21 days after introduction of the arrhythmic stimulus (128).

## The Translational Role of Large Animal Models of HF: A Critical Part of Clinical Success?

**Figure 2:**
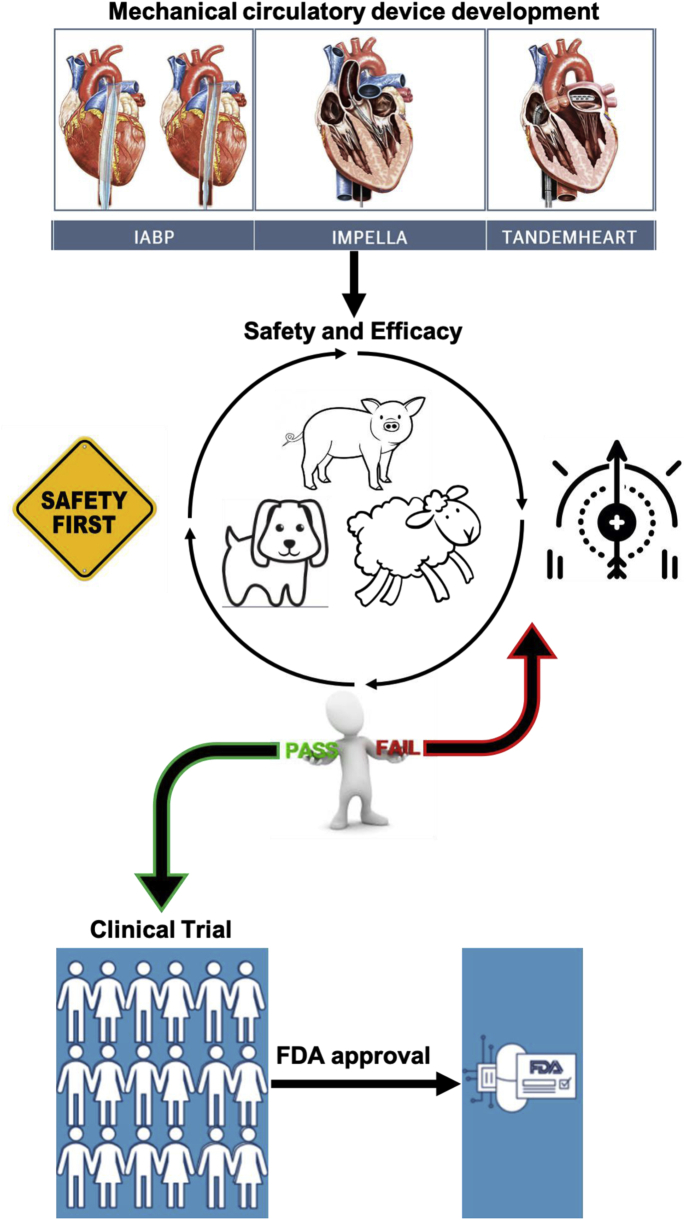
Proposed Translational Flow for Development and Testing of New Therapies and Devices to Clinical Trials and FDA Approval Continuous testing and evaluation of safety and/or efficacy during the preclinical phase is a proposed pathway to improving clinical success. (Intra-aortic balloon pump [IABP], Impella, and TandemHeart pictures from were originally published in Atkinson et al. [170]). FDA = Food and Drug Administration.

Pre-clinical large animal models of cardiovascular disease are an essential, but arguably underused translational bridge to the development and testing of new therapies and devices before clinical trials (129). Clinical attrition rates for research and development range from 80% to 97%, with clinical segments of the overall studies accounting for 73% of the total cost of bringing the therapeutic to market (130). This high rate of failure occurs despite preclinical attrition rates of only 35%, which suggests high levels of success in animal research may be “fool’s gold.” Several factors have been proposed regarding the failure of animal research to translate clinically, including overoptimistic conclusions inferred from methodologically flawed animal studies, animal models that do not adequately reflect human disease, and neutral or negative outcomes in animal studies that are more likely to remain unpublished (131,132). Together, these factors contribute to a likelihood of an approval rate of approximately 7% for cardiovascular drugs as assessed from phase I clinical trials to official authorization of new drug and/or biologic license application (133). Currently, >60% of the time and money required for the successful approval of a new device or therapeutic is spent during human clinical trials (134). Given the high rate of failure during this phase, studies have suggested that an increase in the amount of time and money spent on candidate selection, safety, and efficacy in the preclinical phase could improve human translation both physiologically and economically (134). Recent viewpoints have proposed follow-up times of ≥1 year in large animal studies to better assess endpoint success pre-clinically before moving to phase I (129). Large animal models of pathophysiology are also necessary to improve translation practically by: 1) facilitating testing of clinical delivery, imaging, and support devices; 2) providing valuable toxicology and biodistribution information; and 3) providing relevant physiological inputs that can guide computational and omics-based assessment of clinical risk useful for precision medicine. Supporting data from large animal models is often a critical aspect of the Investigational New Drug or Device Exemption process that leads to FDA approval. Thus, the following section highlights recent clinical trials that have used preclinical studies as part of the development process for new HF therapeutic support devices (Figure 2).

The FDA evaluates, regulates, and approves various medical products, including mechanical circulatory support devices for patients with HF, such as intra-aortic balloon pumps (IABPs), axial flow pumps, and left atrial-to-femoral arterial ventricular assist devices. These devices mechanically assist the myocardium, providing short-term systemic hemodynamic support and minimizing myocardial workload during ischemic events complicated by cardiogenic shock or during high-risk PCI procedures (135, 136, 137). IABPs were previously considered class I treatment for acute MI complicated by cardiogenic shock (138, 139, 140) but are currently recommended as class II treatment (141,142). Although widely used in patients with HF, IABPs have hemodynamic and surgical limitations (143, 144, 145). As a result, new mechanical circulatory support devices have been developed for clinical use. These devices support cardiac function by collecting blood from: 1) the LV and delivering it into the ascending aorta (135,146,147); or 2) the left atrium and delivering the blood to the femoral artery by centrifugal bypass (135,148, 149, 150). Two of these devices are the FDA-approved TandemHeart (left atrial-to-femoral arterial ventricular assist device) (LivaNova PLC, London, United Kingdom) and Impella (Axial flow pump) LV support systems (Abiomed Inc., Danvers, Massachusetts). TandemHeart supports systemic hemodynamics by pumping blood up to 4 l/min, whereas several Impella models (Impella CP, Impella 2.5, Impella 5.0/LD, and Impella RP) provide flow ranging from 2.5 to 5.0 l/min. The efficacy and safety of these systems has been tested in several large animal models of HF, which are reported adjacent to their associated clinical trials in Table 2.

**Table 2:** Summary of Clinical Trials for Mechanical Circulatory Support Devices and Associated Preclinical Large Animal Studies

Mechanical Circulatory Support Device/Clinical Trial Name	Clinical Trial Identifier	Clinical Trial status	Patients	Large Animal Studies
Impella System∗ (2.5; CP; LD; LP 2.5)				
DTU	NCT03000270	Completed	50	Pig (152,156, 157, 158, 159, 160, 161); sheep (162,163)
REVERSE	NCT03431467	Recruiting	96	
PERMIT1	NCT01294267	Completed	20	
RECOVER I	NCT00596726	Completed	17	
PROTECT I	NCT00534859	Completed	28	
CARDSUP	NCT04117230	Recruiting	1500	
Protect Kidney Trial	NCT04321148	Recruiting	224	
ISAR-SHOCK	NCT00417378	Completed	26	
Protect PCI Study	NCT02831881	Recruiting	369	
TandemHeart†				
THEME	NCT02326402	Recruiting	200	Pig (151, 152, 153, 154, 155)
ANCHOR	NCT04184635	Active/Not yet recruiting	400	
CentriMag Circulatory‡				
Failure-to-Wean	NCT00819793	Completed	32	Sheep (164, 165, 166); pig (167)
CMagRVAS	NCT01568424	Completed	25	

ANCHOR = Assessment of ECMO in Acute Myocardial Infarction Cardiogenic Shock; CARDSUP = Swiss Circulatory Support Registry; CMagRVAS = CentriMag RVAS U.S. Post-approval Study Protocol; DTU = Door To Unloading With IMPELLA CP System in Acute Myocardial Infarction - Safety and Feasibility Study; Failure-to-Wean = CentriMag Ventricular Assist System in Treating Failure-to-Wean From Cardiopulmonary Bypass; ISAR-SHOCK = Efficacy Study of LV Assist Device to Treat Patients With Cardiogenic Shock; PERMIT1 = Percutaneous Hemodynamic Support With Impella 2.5 During Scar-related Ventricular Tachycardia Ablation; PROTECT I = A Prospective Feasibility Trial Investigating the Use of IMPELLA RECOVER LP 2.5 System in Patients Undergoing High Risk PCI; RECOVER I = RECOVER I Impella RECOVER LP/LD 5.0 Support System Safety and Feasibility Study; REVERSE = Impella CP With VA ECMO for Cardiogenic Shock; THEME = TandemHeart Experiences and Methods.

∗ Food and Drug Administration (FDA) approval: P140003 and 510K-K063723.

† FDA approval: 510(k)-K110493.

‡ FDA approval: recalled.

The impact of TandemHeart on hemodynamics and cardiac morphology has been investigated in porcine models of acute MI or ventricular arrhythmia (151, 152, 153, 154, 155). For example, TandemHeart was implanted during LCx occlusion (30 min) and effectively unloaded the LV while maintaining systemic pressure, which was evident via decreased stroke volume, end-diastolic volume, and EDP (151). Impella devices have also been investigated in swine (152,156, 157, 158, 159, 160, 161) and ovine (162,163) models of adult and pediatric HF. Using their recently described combination I/R−coronary microembolization protocol in Yorkshire swine, Watanabe et al. (159) examined mechanical LV unloading 2 weeks post-MI using the Impella CP. After 2 h of circulatory support, Impella decreased LV end-diastolic volume and EDP, maintained peripheral vascular pressure, and increased coronary vascular perfusion in the infarct area. An interesting comparison between Impella and TandemHeart was assessed in Yorkshire swine (76 kg) subjected to acute MI induced by occlusion of the LCx for 2 h followed by 30 min of reperfusion (152). At comparable flow rates, TandemHeart decreased LV preload (end-diastolic volume), stroke volume, and contractility (dP/dt_max_, stroke work, pre-load recruitable stroke work) to a greater extent than the Impella.

Although the preceding studies reflect successful translational interactions and outcomes between large animal and human studies, the process is not infallible. One example is the CentriMag Circulatory ventricular assist device (Abbott Laboratories, Abbott Park, Illinois), which was recalled due to a calibrating system error linked to electromagnetic interference, which caused the device to stop (https://www.fda.gov/medical-devices/medical-device-recalls/abbott-recalls-centrimag-circulatory-support-system-motor-due-pump-and-motor-issues). Difficulties with the device occurred after FDA approval, despite several preclinical studies in both sheep (164, 165, 166) and pigs (167), 3 registered clinical trials (Table 2) (CentriMag Ventricular Assist System in Treating Failure-to-Wean From Cardiopulmonary Bypass; NCT00819793 [completed]; CentriMag Ventricular Assist System in Treating Failure-to-Wean From Cardiopulmonary Bypass for Pediatric Patients; NCT01171950 [withdrawn]; and CentriMag RVAS U.S. Post-approval Study Protocol [CMagRVAS]; NCT01568424 [completed]), and a multicenter study that showed short-term support with low incidence of device-related complications and no device failure in cardiogenic shock patients 30 days after CentriMag implantation (168). These findings are not meant to undermine the importance of integrating preclinical and clinical studies for assessing safety and feasibility during development, but rather to highlight that work still remains regarding optimization of current systems that facilitate testing and/or approval of new therapies and devices.

## Conclusions

Preclinical large animal models play a critical and expanding role in translating basic science findings to the development and clinical approval of novel cardiovascular therapeutics. As recently examined in mice (169), researchers are similarly encouraged to consider the strengths and weaknesses of large animal models, including specific breeds, comorbidities, disease modifiers, and overall study goals such as acute or chronic outcomes. Space, cost, and competencies should also be taken into consideration given differences in U.S. Department of Agriculture requirements for large animal housing and surgical expertise can vary greatly between large animal species. Increasing the use of large animal models of HF holds significant potential to identify novel mechanisms underlying the HF condition, to provide valuable information regarding the safety and efficacy of new therapies, and to improve physiological and economical translation of animal research to the successful treatment of human HF.

## References

[1] Benjamin E.J., Muntner P., Alonso A. (2019). Heart disease and stroke statistics-2019 update: a report from the American Heart Association. Circulation.

[2] Yancy C.W., Jessup M., Bozkurt B. (2017). 2017 ACC/AHA/HFSA focused update of the 2013 ACCF/AHA guideline for the management of heart failure: a report of the American College of Cardiology/American Heart Association Task Force on Clinical Practice Guidelines and the Heart Failure Society of America. J Am Coll Cardiol.

[3] Yancy C.W., Jessup M., Bozkurt B. (2013). 2013 ACCF/AHA guideline for the management of heart failure: a report of the American College of Cardiology Foundation/American Heart Association Task Force on Practice Guidelines. J Am Coll Cardiol.

[4] Ponikowski P., Voors A.A., Anker S.D. (2016). 2016 ESC Guidelines for the diagnosis and treatment of acute and chronic heart failure: the task force for the diagnosis and treatment of acute and chronic heart failure of the European Society of Cardiology (ESC) developed with the special contribution of the Heart Failure Association (HFA) of the ESC. Eur Heart J.

[5] Hughes H.C. (1986). Swine in cardiovascular research. Lab Anim Sci.

[6] Yuan B.X., Ardell J.L., Hopkins D.A., Losier A.M., Armour J.A. (1994). Gross and microscopic anatomy of the canine intrinsic cardiac nervous system. Anat Rec.

[7] Crick S.J., Sheppard M.N., Ho S.Y., Gebstein L., Anderson R.H. (1998). Anatomy of the pig heart: comparisons with normal human cardiac structure. J Anat.

[8] Lelovas P.P., Kostomitsopoulos N.G., Xanthos T.T. (2014). A comparative anatomic and physiologic overview of the porcine heart. J Am Assoc Lab Anim Sci.

[9] Milani-Nejad N., Janssen P.M. (2014). Small and large animal models in cardiac contraction research: advantages and disadvantages. Pharmacol Ther.

[10] Markovitz L.J., Savage E.B., Ratcliffe M.B. (1989). Large animal model of left ventricular aneurysm. Ann Thorac Surg.

[11] Sorop O., van de Wouw J., Chandler S. (2020). Experimental animal models of coronary microvascular dysfunction. Cardiovasc Res.

[12] Douglas W.R. (1977). Of pigs and men and research: a review of appreciation, and the logic of the pig in human medical research. Space Life Sci.

[13] Armstrong R.B., Delp M.D., Goljan E.F., Laughlin M.H. (1987). Distribution of blood flow in muscles of miniature swine during exercise. J Appl Physiol (1985).

[14] Braunwald E. (2018). Aortic stenosis: then and now. Circulation.

[15] Carabello B.A. (2006). Aortic stenosis: from pressure overload to heart failure. Heart Fail Clin.

[16] Yarbrough W.M., Mukherjee R., Ikonomidis J.S., Zile M.R., Spinale F.G. (2012). Myocardial remodeling with aortic stenosis and after aortic valve replacement: mechanisms and future prognostic implications. J Thorac Cardiovasc Surg.

[17] Houser S.R., Margulies K.B., Murphy A.M. (2012). Animal models of heart failure: a scientific statement from the American Heart Association. Circ Res.

[18] Ross J., Braunwald E. (1968). Aortic stenosis. Circulation.

[19] Mohammed S.F., Hussain S., Mirzoyev S.A., Edwards W.D., Maleszewski J.J., Redfield M.M. (2015). Coronary microvascular rarefaction and myocardial fibrosis in heart failure with preserved ejection fraction. Circulation.

[20] Heusch G., Libby P., Gersh B. (2014). Cardiovascular remodelling in coronary artery disease and heart failure. Lancet.

[21] Gong G., Liu J., Liang P. (2003). Oxidative capacity in failing hearts. Am J Physiol Heart Circ Physiol.

[22] Ye Y., Gong G., Ochiai K., Liu J., Zhang J. (2001). High-energy phosphate metabolism and creatine kinase in failing hearts: a new porcine model. Circulation.

[23] Wang X., Hu Q., Mansoor A. (2006). Bioenergetic and functional consequences of stem cell-based VEGF delivery in pressure-overloaded swine hearts. Am J Physiol Heart Circ Physiol.

[24] Fleenor B.S., Ouyang A., Olver T.D. (2018). Saxagliptin prevents increased coronary vascular stiffness in aortic-banded mini swine. Hypertension.

[25] Hiemstra J.A., Lee D.I., Chakir K. (2016). Saxagliptin and tadalafil differentially alter cyclic guanosine monophosphate (cGMP) signaling and left ventricular function in aortic-banded mini-swine. J Am Heart Assoc.

[26] Hiemstra J.A., Veteto A.B., Lambert M.D. (2018). Chronic low-intensity exercise attenuates cardiomyocyte contractile dysfunction and impaired adrenergic responsiveness in aortic-banded mini-swine. J Appl Physiol (1985).

[27] Olver T.D., Edwards J.C., Ferguson B.S. (2018). Chronic interval exercise training prevents BKCa channel-mediated coronary vascular dysfunction in aortic-banded miniswine. J Appl Physiol (1985).

[28] Hayward G.C., LeBlanc P.J., Emter C.A. (2019). Female sex hormones and cardiac pressure overload independently contribute to the cardiogenic dementia profile in Yucatan miniature swine. Front Cardiovasc Med.

[29] Olver T.D., Hiemstra J.A., Edwards J.C., Ferguson B.S., Laughlin M.H., Emter C.A. (2017). The protective role of sex hormones in females and exercise prehabilitation in males on sternotomy-induced cranial hypoperfusion in aortic banded mini-swine. J Appl Physiol (1985).

[30] Olver T.D., Hiemstra J.A., Edwards J.C. (2017). Loss of female sex hormones exacerbates cerebrovascular and cognitive dysfunction in aortic banded miniswine through a neuropeptide Y-Ca(2+)-activated potassium channel-nitric oxide mediated mechanism. J Am Heart Assoc.

[31] Olver T.D., Klakotskaia D., Ferguson B.S. (2016). Carotid artery vascular mechanics serve as biomarkers of cognitive dysfunction in aortic-banded miniature swine that can be treated with an exercise intervention. J Am Heart Assoc.

[32] Ouyang A., Olver T.D., Emter C.A., Fleenor B.S. (2019). Chronic exercise training prevents coronary artery stiffening in aortic-banded miniswine: role of perivascular adipose-derived advanced glycation end products. J Appl Physiol (1985).

[33] Ishikawa K., Aguero J., Oh J.G. (2015). Increased stiffness is the major early abnormality in a pig model of severe aortic stenosis and predisposes to congestive heart failure in the absence of systolic dysfunction. J Am Heart Assoc.

[34] Yarbrough W.M., Mukherjee R., Stroud R.E. (2012). Progressive induction of left ventricular pressure overload in a large animal model elicits myocardial remodeling and a unique matrix signature. J Thorac Cardiovasc Surg.

[35] Olver T.D., Edwards J.C., Jurrissen T.J. (2019). Western diet-fed, aortic-banded Ossabaw swine: a preclinical model of cardio-metabolic heart failure. J Am Coll Cardiol Basic Transl Sci.

[36] Bratz I.N., Dick G.M., Tune J.D. (2008). Impaired capsaicin-induced relaxation of coronary arteries in a porcine model of the metabolic syndrome. Am J Physiol Heart Circ Physiol.

[37] Dyson M.C., Alloosh M., Vuchetich J.P., Mokelke E.A., Sturek M. (2006). Components of metabolic syndrome and coronary artery disease in female Ossabaw swine fed excess atherogenic diet. Comp Med.

[38] Neeb Z.P., Edwards J.M., Alloosh M., Long X., Mokelke E.A., Sturek M. (2010). Metabolic syndrome and coronary artery disease in Ossabaw compared with Yucatan swine. Comp Med.

[39] Padilla J., Jenkins N.T., Lee S. (2013). Vascular transcriptional alterations produced by juvenile obesity in Ossabaw swine. Physiol Genomics.

[40] Panasevich M.R., Meers G.M., Linden M.A. (2018). High-fat, high-fructose, high-cholesterol feeding causes severe NASH and cecal microbiota dysbiosis in juvenile Ossabaw swine. Am J Physiol Endocrinol Metab.

[41] Toedebusch R.G., Roberts M.D., Wells K.D. (2014). Unique transcriptomic signature of omental adipose tissue in Ossabaw swine: a model of childhood obesity. Physiol Genomics.

[42] Vieira-Potter V.J., Lee S., Bayless D.S. (2015). Disconnect between adipose tissue inflammation and cardiometabolic dysfunction in Ossabaw pigs. Obesity (Silver Spring).

[43] Olver T.D., Grunewald Z.I., Jurrissen T.J. (2018). Microvascular insulin resistance in skeletal muscle and brain occurs early in the development of juvenile obesity in pigs. Am J Physiol Regul Integr Comp Physiol.

[44] Shah S.J., Borlaug B.A., Kitzman D.W. (2020). Research priorities for heart failure with preserved ejection fraction: National Heart, Lung, and Blood Institute working group summary. Circulation.

[45] Shah S.J., Kitzman D.W., Borlaug B.A. (2016). Phenotype-specific treatment of heart failure with preserved ejection fraction: a multiorgan roadmap. Circulation.

[46] Paulus W.J., Tschope C. (2013). A novel paradigm for heart failure with preserved ejection fraction: comorbidities drive myocardial dysfunction and remodeling through coronary microvascular endothelial inflammation. J Am Coll Cardiol.

[47] Aoyagi T., Mirsky I., Flanagan M.F., Currier J.J., Colan S.D., Fujii A.M. (1992). Myocardial function in immature and mature sheep with pressure-overload hypertrophy. Am J Physiol.

[48] Aoyagi T., Fujii A.M., Flanagan M.F. (1993). Transition from compensated hypertrophy to intrinsic myocardial dysfunction during development of left ventricular pressure-overload hypertrophy in conscious sheep. Systolic dysfunction precedes diastolic dysfunction. Circulation.

[49] Moorjani N., Catarino P., El-Sayed R. (2003). A pressure overload model to track the molecular biology of heart failure. Eur J Cardiothorac Surg.

[50] Moorjani N., Catarino P., Trabzuni D. (2007). Upregulation of Bcl-2 proteins during the transition to pressure overload-induced heart failure. Int J Cardiol.

[51] Walther T., Falk V., Binner C. (2000). Experimental aortic stenosis and corresponding left ventricular hypertrophy in sheep. J Invest Surg.

[52] Quttainah M., Al-Hejailan R., Saleh S. (2015). Progression of matrixin and cardiokine expression patterns in an ovine model of heart failure and recovery. Int J Cardiol.

[53] Hearse D.J. (2000). Species variation in the coronary collateral circulation during regional myocardial ischaemia: a critical determinant of the rate of evolution and extent of myocardial infarction. Cardiovasc Res.

[54] Bache R.J., Alyono D., Sublett E., Dai X.Z. (1986). Myocardial blood flow in left ventricular hypertrophy developing in young and adult dogs. Am J Physiol.

[55] Dellsperger K.C., Marcus M.L. (1990). Effects of left ventricular hypertrophy on the coronary circulation. Am J Cardiol.

[56] Gaasch W.H., Zile M.R., Hoshino P.K., Apstein C.S., Blaustein A.S. (1989). Stress-shortening relations and myocardial blood flow in compensated and failing canine hearts with pressure-overload hypertrophy. Circulation.

[57] Sasayama S., Ross J., Franklin D., Bloor C.M., Bishop S., Dilley R.B. (1976). Adaptations of the left ventricle to chronic pressure overload. Circ Res.

[58] Vatner D.E., Homcy C.J., Sit S.P., Manders W.T., Vatner S.F. (1984). Effects of pressure overload, left ventricular hypertrophy on beta-adrenergic receptors, and responsiveness to catecholamines. J Clin Invest.

[59] Goldblatt H., Lynch J., Hanzal R.F., Summerville W.W. (1934). Studies on experimental hypertension: I. The production of persistent elevation of systolic blood pressure by means of renal ischemia. J Exp Med.

[60] Page I.H. (1939). A method for producing persistent hypertension by cellophane. Science.

[61] Shapiro B.P., Owan T.E., Mohammed S. (2008). Mineralocorticoid signaling in transition to heart failure with normal ejection fraction. Hypertension.

[62] Munagala V.K., Hart C.Y., Burnett J.C., Meyer D.M., Redfield M.M. (2005). Ventricular structure and function in aged dogs with renal hypertension: a model of experimental diastolic heart failure. Circulation.

[63] Maniu C.V., Meyer D.M., Redfield M.M. (2002). Hemodynamic and humoral effects of vasopeptidase inhibition in canine hypertension. Hypertension.

[64] Hart C.Y., Meyer D.M., Tazelaar H.D. (2001). Load versus humoral activation in the genesis of early hypertensive heart disease. Circulation.

[65] Sorop O., Heinonen I., van Kranenburg M. (2018). Multiple common comorbidities produce left ventricular diastolic dysfunction associated with coronary microvascular dysfunction, oxidative stress, and myocardial stiffening. Cardiovasc Res.

[66] Schwarzl M., Hamdani N., Seiler S. (2015). A porcine model of hypertensive cardiomyopathy: implications for heart failure with preserved ejection fraction. Am J Physiol Heart Circ Physiol.

[67] Reiter U., Reiter G., Manninger M. (2016). Early-stage heart failure with preserved ejection fraction in the pig: a cardiovascular magnetic resonance study. J Cardiovasc Magn Reson.

[68] Thygesen K., Alpert J.S., White H.D. (2007). Universal definition of myocardial infarction. J Am Coll Cardiol.

[69] Alpert J.S., Thygesen K., Antman E., Bassand J.P. (2000). Myocardial infarction redefined--a consensus document of The Joint European Society of Cardiology/American College of Cardiology Committee for the redefinition of myocardial infarction. J Am Coll Cardiol.

[70] Thygesen K., Alpert J.S., Jaffe A.S. (2018). Fourth universal definition of myocardial infarction (2018). J Am Coll Cardiol.

[71] Jennings R.B., Ganote C.E. (1974). Structural changes in myocardium during acute ischemia. Circ Res.

[72] Reimer K.A., Jennings R.B., Tatum A.H. (1983). Pathobiology of acute myocardial ischemia: metabolic, functional and ultrastructural studies. Am J Cardiol.

[73] Davidson S.M., Ferdinandy P., Andreadou I. (2019). Multitarget strategies to reduce myocardial ischemia/reperfusion injury: JACC review topic of the week. J Am Coll Cardiol.

[74] Hausenloy D.J., Yellon D.M. (2013). Myocardial ischemia-reperfusion injury: a neglected therapeutic target. J Clin Invest.

[75] Bikou O., Watanabe S., Hajjar R.J., Ishikawa K. (2018). A pig model of myocardial infarction: catheter-based approaches. Methods Mol Biol.

[76] Bolli R., Zhu W.X., Thornby J.I., O'Neill P.G., Roberts R. (1988). Time course and determinants of recovery of function after reversible ischemia in conscious dogs. Am J Physiol.

[77] Verma S., Fedak P.W., Weisel R.D. (2002). Fundamentals of reperfusion injury for the clinical cardiologist. Circulation.

[78] Erbel R., Heusch G. (2000). Coronary microembolization. J Am Coll Cardiol.

[79] Heusch G., Skyschally A., Kleinbongard P. (2018). Coronary microembolization and microvascular dysfunction. Int J Cardiol.

[80] Falk E. (1985). Unstable angina with fatal outcome: dynamic coronary thrombosis leading to infarction and/or sudden death. Autopsy evidence of recurrent mural thrombosis with peripheral embolization culminating in total vascular occlusion. Circulation.

[81] Topol E.J., Yadav J.S. (2000). Recognition of the importance of embolization in atherosclerotic vascular disease. Circulation.

[82] Heusch G., Kleinbongard P., Bose D. (2009). Coronary microembolization: from bedside to bench and back to bedside. Circulation.

[83] Tamis-Holland J.E., Jneid H., Reynolds H.R. (2019). Contemporary diagnosis and management of patients with myocardial infarction in the absence of obstructive coronary artery disease: a scientific statement from the American Heart Association. Circulation.

[84] Mukherjee D. (2019). Myocardial infarction with nonobstructive coronary arteries: a call for individualized treatment. J Am Heart Assoc.

[85] Pasupathy S., Tavella R., Beltrame J.F. (2016). The what, when, who, why, how and where of myocardial infarction with non-obstructive coronary arteries (MINOCA). Circ J.

[86] Saku K., Kakino T., Arimura T. (2018). Left ventricular mechanical unloading by total support of Impella in myocardial infarction reduces infarct size, preserves left ventricular function, and prevents subsequent heart failure in dogs. Circ Heart Fail.

[87] Ishikawa K., Aguero J., Tilemann L. (2014). Characterizing preclinical models of ischemic heart failure: differences between LAD and LCx infarctions. Am J Physiol Heart Circ Physiol.

[88] Pleger S.T., Shan C., Ksienzyk J. (2011). Cardiac AAV9-S100A1 gene therapy rescues post-ischemic heart failure in a preclinical large animal model. Sci Transl Med.

[89] Charles C.J., Elliott J.M., Nicholls M.G., Rademaker M.T., Richards M. (2000). Myocardial infarction with and without reperfusion in sheep: early cardiac and neurohumoral changes. Clin Sci (Lond).

[90] Leshnower B.G., Sakamoto H., Hamamoto H., Zeeshan A., Gorman J.H., Gorman R.C. (2007). Progression of myocardial injury during coronary occlusion in the collateral-deficient heart: a non-wavefront phenomenon. Am J Physiol Heart Circ Physiol.

[91] Bowen F.W., Hattori T., Narula N. (2001). Reappearance of myocytes in ovine infarcts produced by six hours of complete ischemia followed by reperfusion. Ann Thorac Surg.

[92] Youngblood B.L., LaRose A., Geist B. (2019). Myocardial infarction model in a sheep. J Pharmacol Toxicol Methods.

[93] van der Velden J., Merkus D., Klarenbeek B.R. (2004). Alterations in myofilament function contribute to left ventricular dysfunction in pigs early after myocardial infarction. Circ Res.

[94] Zeng L., Hu Q., Wang X. (2007). Bioenergetic and functional consequences of bone marrow-derived multipotent progenitor cell transplantation in hearts with postinfarction left ventricular remodeling. Circulation.

[95] Zhang J., Wilke N., Wang Y. (1996). Functional and bioenergetic consequences of postinfarction left ventricular remodeling in a new porcine model. MRI and 31 P-MRS study. Circulation.

[96] O'Konski M.S., White F.C., Longhurst J., Roth D., Bloor C.M. (1987). Ameroid constriction of the proximal left circumflex coronary artery in swine. A model of limited coronary collateral circulation. Am J Cardiovasc Pathol.

[97] Roth D.M., Maruoka Y., Rogers J., White F.C., Longhurst J.C., Bloor C.M. (1987). Development of coronary collateral circulation in left circumflex Ameroid-occluded swine myocardium. Am J Physiol.

[98] Sassoon D.J., Tune J.D., Mather K.J. (2017). Glucagon-like peptide 1 receptor activation augments cardiac output and improves cardiac efficiency in obese swine after myocardial infarction. Diabetes.

[99] Elmadhun N.Y., Lassaletta A.D., Chu L.M., Sellke F.W. (2013). Metformin alters the insulin signaling pathway in ischemic cardiac tissue in a swine model of metabolic syndrome. J Thorac Cardiovasc Surg.

[100] Lassaletta A.D., Chu L.M., Robich M.P. (2012). Overfed Ossabaw swine with early stage metabolic syndrome have normal coronary collateral development in response to chronic ischemia. Basic Res Cardiol.

[101] Chekanov V., Akhtar M., Tchekanov G. (2003). Transplantation of autologous endothelial cells induces angiogenesis. Pacing Clin Electrophysiol.

[102] Charles C.J., Elliott J.M., Nicholls M.G., Rademaker M.T., Richards A.M. (2003). Natriuretic peptides maintain sodium homoeostasis during chronic volume loading post-myocardial infarction in sheep. Clin Sci (Lond).

[103] Franciosa J.A., Heckel R., Limas C., Cohn J.N. (1980). Progressive myocardial dysfunction associated with increased vascular resistance. Am J Physiol.

[104] Sabbah H.N., Stein P.D., Kono T. (1991). A canine model of chronic heart failure produced by multiple sequential coronary microembolizations. Am J Physiol.

[105] Carlsson M., Wilson M., Martin A.J., Saeed M. (2009). Myocardial microinfarction after coronary microembolization in swine: MR imaging characterization. Radiology.

[106] Hegyi B., Bossuyt J., Griffiths L.G. (2018). Complex electrophysiological remodeling in postinfarction ischemic heart failure. Proc Natl Acad Sci U S A.

[107] Moller-Helgestad O.K., Ravn H.B., Moller J.E. (2018). Large porcine model of profound acute ischemic cardiogenic shock. Methods Mol Biol.

[108] Huang Y., Kawaguchi O., Zeng B. (1997). A stable ovine congestive heart failure model. A suitable substrate for left ventricular assist device assessment. ASAIO J.

[109] Ikeda Y., Yutani C., Huang Y. (2001). Histological remodeling in an ovine heart failure model resembles human ischemic cardiomyopathy. Cardiovasc Pathol.

[110] Monreal G., Gerhardt M.A., Kambara A., Abrishamchian A.R., Bauer J.A., Goldstein A.H. (2004). Selective microembolization of the circumflex coronary artery in an ovine model: dilated, ischemic cardiomyopathy and left ventricular dysfunction. J Card Fail.

[111] Bikou O., Tharakan S., Yamada K.P. (2019). A novel large animal model of thrombogenic coronary microembolization. Front Cardiovasc Med.

[112] Masarone D., Limongelli G., Rubino M. (2017). Management of arrhythmias in heart failure. J Cardiovasc Dev Dis.

[113] Huizar J.F., Ellenbogen K.A., Tan A.Y., Kaszala K. (2019). Arrhythmia-induced cardiomyopathy: JACC state-of-the-art review. J Am Coll Cardiol.

[114] January C.T., Wann L.S., Alpert J.S. (2014). 2014 AHA/ACC/HRS guideline for the management of patients with atrial fibrillation: a report of the American College of Cardiology/American Heart Association Task Force on Practice Guidelines and the Heart Rhythm Society. J Am Coll Cardiol.

[115] Schotten U., Verheule S., Kirchhof P., Goette A. (2011). Pathophysiological mechanisms of atrial fibrillation: a translational appraisal. Physiol Rev.

[116] Iwasaki Y.K., Nishida K., Kato T., Nattel S. (2011). Atrial fibrillation pathophysiology: implications for management. Circulation.

[117] Whipple G.H.S.L., Woodman E.G., Theophilis C., Friedman S. (1962). Reversible congestive heart failure due to chronic rapid stimulation of the normal heart. Proc N Engl Cardiovasc Soc.

[118] Packer D.L., Bardy G.H., Worley S.J. (1986). Tachycardia-induced cardiomyopathy: a reversible form of left ventricular dysfunction. Am J Cardiol.

[119] Wilson J.R., Douglas P., Hickey W.F. (1987). Experimental congestive heart failure produced by rapid ventricular pacing in the dog: cardiac effects. Circulation.

[120] Hendrick D.A., Smith A.C., Kratz J.M., Crawford F.A., Spinale F.G. (1990). The pig as a model of tachycardia and dilated cardiomyopathy. Lab Anim Sci.

[121] Spinale F.G., Hendrick D.A., Crawford F.A., Smith A.C., Hamada Y., Carabello B.A. (1990). Chronic supraventricular tachycardia causes ventricular dysfunction and subendocardial injury in swine. Am J Physiol.

[122] Fitzpatrick M.A., Nicholls M.G., Espiner E.A., Ikram H., Bagshaw P., Yandle T.G. (1989). Neurohumoral changes during onset and offset of ovine heart failure: role of ANP. Am J Physiol.

[123] Rademaker M.T., Charles C.J., Espiner E.A., Frampton C.M., Nicholls M.G., Richards A.M. (1996). Natriuretic peptide responses to acute and chronic ventricular pacing in sheep. Am J Physiol.

[124] Mollmann H., Voss S., Nef H.M. (2009). Desynchronization: a novel model to induce heart failure. Thorac Cardiovasc Surg.

[125] Dosdall D.J., Ranjan R., Higuchi K. (2013). Chronic atrial fibrillation causes left ventricular dysfunction in dogs but not goats: experience with dogs, goats, and pigs. Am J Physiol Heart Circ Physiol.

[126] Bauer A., McDonald A.D., Donahue J.K. (2004). Pathophysiological findings in a model of persistent atrial fibrillation and severe congestive heart failure. Cardiovasc Res.

[127] Avitall B., Bi J., Mykytsey A., Chicos A. (2008). Atrial and ventricular fibrosis induced by atrial fibrillation: evidence to support early rhythm control. Heart Rhythm.

[128] Byrne M., Kaye D.M., Power J. (2008). The synergism between atrial fibrillation and heart failure. J Card Fail.

[129] Heusch G. (2018). Cardioprotection research must leave its comfort zone. Eur Heart J.

[130] Garner J.P. (2014). The significance of meaning: why do over 90% of behavioral neuroscience results fail to translate to humans, and what can we do to fix it?. ILAR J.

[131] Hackam D.G., Redelmeier D.A. (2006). Translation of research evidence from animals to humans. JAMA.

[132] van der Worp H.B., Howells D.W., Sena E.S. (2010). Can animal models of disease reliably inform human studies?. PLoS Med.

[133] Hay M., Thomas D.W., Craighead J.L., Economides C., Rosenthal J. (2014). Clinical development success rates for investigational drugs. Nat Biotechnol.

[134] Paul S.M., Mytelka D.S., Dunwiddie C.T. (2010). How to improve R&D productivity: the pharmaceutical industry's grand challenge. Nat Rev Drug Discov.

[135] Gilotra N.A., Stevens G.R. (2014). Temporary mechanical circulatory support: a review of the options, indications, and outcomes. Clin Med Insights Cardiol.

[136] Sarkar K., Kini A.S. (2010). Percutaneous left ventricular support devices. Cardiol Clin.

[137] Vahdatpour C., Collins D., Goldberg S. (2019). Cardiogenic Shock. J Am Heart Assoc.

[138] Thiele H., Zeymer U., Neumann F.J. (2012). Intraaortic balloon support for myocardial infarction with cardiogenic shock. N Engl J Med.

[139] McMurray J.J., Adamopoulos S., Anker S.D. (2012). ESC guidelines for the diagnosis and treatment of acute and chronic heart failure 2012: the task force for the diagnosis and treatment of acute and chronic heart failure 2012 of the European Society of Cardiology. Developed in collaboration with the Heart Failure Association (HFA) of the ESC. Eur Heart J.

[140] Kantrowitz A., Tjonneland S., Freed P.S., Phillips S.J., Butner A.N., Sherman J.L. (1968). Initial clinical experience with intraaortic balloon pumping in cardiogenic shock. JAMA.

[141] O'Gara P.T., Kushner F.G., Ascheim D.D. (2013). 2013 ACCF/AHA guideline for the management of ST-elevation myocardial infarction: a report of the American College of Cardiology Foundation/American Heart Association Task Force on Practice Guidelines. J Am Coll Cardiol.

[142] Steg P.G., James S.K., Task Force on the management of ST-segment elevation acute myocardial infarction of the European Society of Cardiology (ESC) (2012). ESC guidelines for the management of acute myocardial infarction in patients presenting with ST-segment elevation. Eur Heart J.

[143] Thiele H., Lauer B., Hambrecht R., Boudriot E., Cohen H.A., Schuler G. (2001). Reversal of cardiogenic shock by percutaneous left atrial-to-femoral arterial bypass assistance. Circulation.

[144] Thiele H., Ohman E.M., Desch S., Eitel I., de Waha S. (2015). Management of cardiogenic shock. Eur Heart J.

[145] Parissis H., Graham V., Lampridis S., Lau M., Hooks G., Mhandu P.C. (2016). IABP: history-evolution-pathophysiology-indications: what we need to know. J Cardiothorac Surg.

[146] O'Neill W.W., Kleiman N.S., Moses J. (2012). A prospective, randomized clinical trial of hemodynamic support with Impella 2.5 versus intra-aortic balloon pump in patients undergoing high-risk percutaneous coronary intervention: the PROTECT II study. Circulation.

[147] Ouweneel D.M., Engstrom A.E., Sjauw K.D. (2016). Experience from a randomized controlled trial with Impella 2.5 versus IABP in STEMI patients with cardiogenic pre-shock. Lessons learned from the IMPRESS in STEMI trial. Int J Cardiol.

[148] Kapur N.K., Paruchuri V., Urbano-Morales J.A. (2013). Mechanically unloading the left ventricle before coronary reperfusion reduces left ventricular wall stress and myocardial infarct size. Circulation.

[149] Kar B., Adkins L.E., Civitello A.B. (2006). Clinical experience with the TandemHeart percutaneous ventricular assist device. Tex Heart Inst J.

[150] Kloner R.A. (2013). Can myocardial infarct size be reduced by mechanically unloading the left ventricle?. Circulation.

[151] Esposito M.L., Shah N., Dow S. (2016). Distinct effects of left or right atrial cannulation on left ventricular hemodynamics in a swine model of acute myocardial injury. ASAIO J.

[152] Weil B.R., Konecny F., Suzuki G., Iyer V., Canty J.M. (2016). Comparative hemodynamic effects of contemporary percutaneous mechanical circulatory support devices in a porcine model of acute myocardial infarction. J Am Coll Cardiol Intv.

[153] Ostadal P., Mlcek M., Holy F. (2012). Direct comparison of percutaneous circulatory support systems in specific hemodynamic conditions in a porcine model. Circ Arrhythm Electrophysiol.

[154] Lim D.S., Cortese C.J., Loree A.N., Dean D.A., Svitek R.G. (2009). Left ventricular assist via percutaneous transhepatic transseptal cannulation in swine. Catheter Cardiovasc Interv.

[155] Kulat B.T., Russell H.M., Sarwark A.E. (2014). Modified TandemHeart ventricular assist device for infant and pediatric circulatory support. Ann Thorac Surg.

[156] Hammoudi N., Watanabe S., Bikou O. (2019). Speckle-tracking echocardiographic strain analysis reliably estimates degree of acute LV unloading during mechanical LV support by Impella. J Cardiovasc Transl Res.

[157] Ko B., Drakos S.G., Ibrahim H. (2020). Percutaneous mechanical unloading simultaneously with reperfusion induces increased myocardial salvage in experimental acute myocardial infarction. Circ Heart Fail.

[158] Moller-Helgestad O.K., Poulsen C.B., Christiansen E.H., Lassen J.F., Ravn H.B. (2015). Support with intra-aortic balloon pump vs. Impella2.5(R) and blood flow to the heart, brain and kidneys - an experimental porcine model of ischaemic heart failure. Int J Cardiol.

[159] Watanabe S., Fish K., Kovacic J.C. (2018). Left ventricular unloading using an Impella CP improves coronary flow and infarct zone perfusion in ischemic heart failure. J Am Heart Assoc.

[160] Webb M.K., Wang J., Riegel M.S. (2016). Initial experience with the pediatric Impella device: a feasibility study in a porcine model. Catheter Cardiovasc Interv.

[161] Esposito M.L., Zhang Y., Qiao X. (2018). Left ventricular unloading before reperfusion promotes functional recovery after acute myocardial infarction. J Am Coll Cardiol.

[162] Rega F.R., Vantichelen I., Bollen H. (2008). Pediatric heart support with a newly developed catheter based pulsatile 12F rotary blood pump: an animal study. Eur J Cardiothorac Surg.

[163] Wei X., Li T., Hagen B. (2013). Short-term mechanical unloading with left ventricular assist devices after acute myocardial infarction conserves calcium cycling and improves heart function. J Am Coll Cardiol Intv.

[164] Pirbodaghi T., Axiak S., Weber A., Gempp T., Vandenberghe S. (2012). Pulsatile control of rotary blood pumps: Does the modulation waveform matter?. J Thorac Cardiovasc Surg.

[165] Pirbodaghi T., Weber A., Carrel T., Vandenberghe S. (2011). Effect of pulsatility on the mathematical modeling of rotary blood pumps. Artif Organs.

[166] Wang D., Plunkett M., Lynch J., Zhou X., Ballard-Croft C., Zwischenberger J.B. (2011). Wang-Zwische double-lumen cannula leads to total cavopulmonary support in a failing Fontan sheep model. Ann Thorac Surg.

[167] Sanchez-Lorente D., Go T., Jungebluth P. (2010). Single double-lumen venous-venous pump-driven extracorporeal lung membrane support. J Thorac Cardiovasc Surg.

[168] John R., Long J.W., Massey H.T. (2011). Outcomes of a multicenter trial of the Levitronix CentriMag ventricular assist system for short-term circulatory support. J Thorac Cardiovasc Surg.

[169] Ghazalpour A., Rau C.D., Farber C.R. (2012). Hybrid mouse diversity panel: a panel of inbred mouse strains suitable for analysis of complex genetic traits. Mamm Genome.

[170] Atkinson T.M., Ohman E.M., O'Neill W.W., Rab T., Cigarroa J.E. (2016). Interventional Scientific Council of the American College of Cardiology. A practical approach to mechanical circulatory support in patients undergoing percutaneous coronary intervention: an interventional perspective. J Am Coll Cardiol Intv.

